# Host Factors Affecting Generation of Immunity Against Porcine Epidemic Diarrhea Virus in Pregnant and Lactating Swine and Passive Protection of Neonates

**DOI:** 10.3390/pathogens9020130

**Published:** 2020-02-18

**Authors:** Stephanie N. Langel, Qiuhong Wang, Anastasia N. Vlasova, Linda J. Saif

**Affiliations:** 1 Duke Human Vaccine Institute, Duke University Medical Center, Durham, NC 27710, USA; 2 Food Animal Health Research Program, Ohio Agricultural Research and Development Center, College of Food, Agricultural and Environmental Sciences, Department of Veterinary Preventive Medicine, The Ohio State University, Wooster, OH 44691, USA; wang.655@osu.edu (Q.W.); vlasova.1@osu.edu (A.N.V.)

**Keywords:** PEDV, lactogenic immunity, IgA antibodies, gut-mammary gland-secretory IgA axis, pregnancy, lymphocyte trafficking

## Abstract

Porcine epidemic diarrhea virus (PEDV) is a highly virulent re-emerging enteric coronavirus that causes acute diarrhea, dehydration, and up to 100% mortality in neonatal suckling piglets. Despite this, a safe and effective PEDV vaccine against highly virulent strains is unavailable, making PEDV prevention and control challenging. Lactogenic immunity induced via the gut-mammary gland-secretory IgA (sIgA) axis, remains the most promising and effective way to protect suckling piglets from PEDV. Therefore, a successful PEDV vaccine must induce protective maternal IgA antibodies that passively transfer into colostrum and milk. Identifying variables that influence lymphocyte migration and IgA secretion during gestation and lactation is imperative for designing maternal immunization strategies that generate the highest amount of lactogenic immune protection against PEDV in suckling piglets. Because pregnancy-associated immune alterations influence viral pathogenesis and adaptive immune responses in many different species, a better understanding of host immune responses to PEDV in pregnant swine may translate into improved maternal immunization strategies against enteric pathogens for multiple species. In this review, we discuss the role of host factors during pregnancy on antiviral immunity and their implications for generating protective lactogenic immunity in suckling neonates.

## 1. Introduction

The generation of maternal immunity during gestation and lactation provides a dual benefit in protection against infectious agents for the mother-neonatal dyad. This is especially true for swine, whose epitheliochorial placenta inhibits immunoglobulin transfer *in utero* [1]. Therefore, colostrum and milk-derived maternal antibodies and other immune factors are the sole source for immune protection of suckling piglets after birth. Passive lactogenic immunity is achieved through high titers of IgG antibodies in colostrum and a continuous supply of secretory IgA (sIgA) antibodies in colostrum and milk. Specifically, because of their persistence in milk at high titers, sIgA antibodies play a major role in conferring passive lactogenic protection against enteric pathogens in suckling neonates. Therefore, lactogenic immunity remains the most promising and effective way to protect neonatal piglets from a recently re-emerged highly virulent enteric coronavirus, porcine epidemic diarrhea virus (PEDV). High rates of protection were achieved when pregnant sows were orally infected with live virulent PEDV [2]. The increased rates of protection were associated with high titers of sIgA antibodies in colostrum and milk [2]. This demonstrates that protecting suckling piglets from devastating enteric viral pathogens is dependent on efficient trafficking of intestinal IgA^+^ plasmablasts to the mammary gland (MG) and accumulation of sIgA antibodies in milk, defined as the gut-MG-sIgA axis [2,3]. While it is known that the migration of IgA^+^ plasmablasts to the MG depends on the regulation of mucosal homing receptor and chemokine expression, the mechanisms that regulate this process are much less understood. Identifying variables that influence lymphocyte migration during gestation and lactation is imperative for designing maternal immunization strategies that generate the highest amount of lactogenic immune protection against PEDV in suckling piglets. In this review, we will discuss the role of host factors during pregnancy on antiviral immunity and its implications for generating protective lactogenic immunity against PEDV infection in neonatal suckling piglets.

## 2. PEDV: A Re-Emerging Enteric Coronavirus

PEDV is a highly virulent re-emerging enteric coronavirus belonging to the *Alphacoronavirus* genus within the family of *Coronaviridae*. Currently, there are four genera, including *Alphacoronavirus, Betacoronavirus, Gammacoronavirus, and Deltacoronavirus,* within the *Coronaviridae* family. Other alphacoronaviruses include transmissible gastroenteritis virus (TGEV) in swine, feline coronaviruses (FCoV), canine coronaviruses (CCoV), ferret enteric coronavirus (FRECV) and two human coronaviruses NL63 and 229E. Additionally, five human coronaviruses (OC43, SARS-CoV, MERS-CoV, HKU1 and SARS-CoV-2) belong to the *Betacoronavirus* genus. PEDV, like other alphacoronaviruses, is genetically distinct from the new SARS-related SARS-CoV-2 that emerged in December 2019 in Wuhan, China and is currently spreading in humans in China and other countries [4]. There is no evidence that PEDV can infect humans. Interestingly, it is speculated that PEDV, like SARS, MERS and SARS-CoV-2 coronaviruses, may have arisen from a bat reservoir [5,6,7,8,9].

PEDV causes acute diarrhea, dehydration and up to 100% mortality in neonatal piglets [10]. Classical PEDV (CV777) was first isolated in 1976 from the feces of young diarrheic pigs in Belgium and demonstrated to induce diarrhea in swine [11,12]. After its identification, outbreaks were detected throughout Europe, severely affecting nursing piglets [13,14]. Although vaccines were not used against PEDV during this time, high biosecurity standards and relatively low pig density resulted in a low overall economic impact. Eventually PEDV became rare in Europe, with only sporadic outbreaks reported. PEDV was first reported in Asia in 1983 [15] where it spread quickly, causing severe epidemics and the disease to ultimately become endemic [16]. Unlike European PEDV outbreaks, the economic impact of PEDV in Asia was much greater resulting in the development and use of attenuated and inactivated PEDV vaccines. While vaccines based on classical PEDV strains CV777 [17], DR13 [16], and 83P-5 [18] decreased mortality, outbreaks of PED were reported on vaccinated farms suggesting insufficient lactogenic immunity against the highly virulent PEDV, which emerged in China in late 2010 [16,19]. Additionally, live attenuated vaccine (LAV) reversion to virulence or recombination with circulating strains may have contributed to the emergence of highly virulent PEDV strains in Asia [20,21].

Highly virulent PEDV (non-S INDEL) emerged in the US as a new, devastating diarrheal disease in 2013 and spread rapidly, causing substantial economic damage to the US swine industry [5,22]. While PEDV infected swine of all ages, severe clinical signs, including vomiting, diarrhea and dehydration, and high mortality rates (up to 100%) were seen in suckling piglets born to naïve dams [23]. Due to its highly contagious nature, PEDV spreads rapidly through the fecal-oral route after direct or indirect exposure to infected animals or their feces [23]. PEDV may also spread through aerosolized particles [24,25]. Genetically the US PEDV strains resembled the highly virulent strains that emerged in China (China AH2012 and CH/ZMZDY/11) [5,26,27]. Later, a PEDV variant containing insertions and deletions (INDELs) in the S1 subunit of the spike (S) protein was detected and designated as S INDEL PEDV [26,28]. The S INDEL strain is more closely related to the classical (CV777) rather than emerging highly virulent PEDV strains, which are designated as non-S INDEL PEDV and exhibited decreased virulence and mortality in piglets [29]. The S protein of PEDV is key for cellular entry and is the main target of neutralizing antibodies [30]. Considering classical vaccines do not provide cross-protection against highly virulent variant PEDV challenge [31,32,33], it is likely that genetic variation of the S gene contributes to viral virulence [34]. Additionally, co-infections of other enteropathogens, like *Escherichia coli* [35,36], porcine circovirus type 2 [37,38] or rotavirus (RV) [39], may exacerbate PEDV in young and adult swine.

There are multiple factors that need to be considered when developing novel immunization strategies against re-emerging highly virulent PEDV. Indeed, a slower turnover rate of enterocytes [40], decreased natural killer (NK) cell function [41], and absence of memory B and T cells to PEDV [42] make neonatal suckling piglets the most susceptible population to PEDV infection, yet more challenging candidates for PEDV vaccination. Therefore, identifying cost effective strategies to stimulate and enhance trafficking of PEDV IgA antibody secreting cells (ASCs) to the MG and accumulation of PEDV sIgA antibodies in milk of pregnant swine is critical for the lactogenic immune protection of suckling piglets immediately after birth. Because pregnancy-associated immune adaptations influence viral pathogenesis and adaptive immune responses in humans and mice, a better understanding of host immune responses to PEDV in pregnant swine is necessary for the development of maternal immunization strategies and therapeutics that enhance lactogenic immunity.

## 3. Host Immune Response to PEDV

### 3.1. Innate Immunity

The innate immune response serves as the first line of defense against viral infections and can prevent the establishment and spread of infection. Host immune cells quickly react to viral infections by producing interferon (IFN)-α/β (type I IFNs) which induce hundreds of IFN-stimulated genes creating an antiviral host state [43,44,45]. Specifically, viruses contain pathogen-associated molecular patterns (PAMPs) like viral nucleic acids and glycoproteins that are recognized through host cell pattern recognition receptors (PRRs). These include three major classes of PRRs: toll-like receptors (TLRs), retinoic acid-inducible gene 1 (RIG-I)-like receptors (RLRs) and NOD-like receptors (NLRs) [43,46]. Upon stimulation, downstream adaptor molecules and IκB kinase- (IKK-) related kinases upregulate IFN regulatory factor 3 (IRF3), nuclear factor κB (NF-κB), and ATF-2/c-jun, which translocate to the nucleus and stimulate IFN-α/β expression [47,48]. However, type I IFNs are more likely to induce antiviral effects systemically and are less effective at restricting viral replication in mucosal epithelial cells and clearing enteric viral pathogens like PEDV [49]. In contrast, recently identified type III IFNs (IFN-λ) are mainly produced by epithelial cells and are potent inducers of IFN-stimulated genes at the mucosal surface [50,51,52]. Many PEDV proteins have been identified that suppress IFN production, including nsp1, nsp3, nsp5, nsp7, nsp14, nsp15, nsp16, ORF3, E, M and N [53,54,55,56,57,58,59]. For example, nsp3 impairs the ability of downstream IFN activation by preventing post-translational modification of RIG-I [59,60]. Recent evidence demonstrated that both nsp15 and nsp16 are important for suppressing IFN responses. Inactivation of these proteins resulted in enhanced induction of type I and III IFN responses after PEDV infection *in vitro* and viral attenuation *in vivo* [55,56]. PEDV further subverts the host antiviral immune response by inducing STAT1 degradation and epidermal growth factor receptor (EGFR) activation [61], inhibiting the IFN-α signaling pathway [54,62]. Targeting PEDV antagonists of innate immunity is essential for controlling infection and designing effective LAVs. During times of cellular stress, including viral infection, mRNA-containing non-membranous stress granules are formed in the cytoplasm and serve as an antiviral innate immune adaptation that negatively impacts viral replication [63]. Indeed, PEDV induced stress granule formation in Vero-76 cells and knockdown of a key stress granule-resident protein, Ras-GTPase-activating protein (SH3 domain) binding protein 1 (G3BP1), increased PEDV replication and proinflammatory cytokine secretion [64]. Consequently, when G3BP1 was overexpressed in Vero-76 cells, PEDV replication decreased suggesting that stress granules play an antiviral role during PEDV infection. Additionally, low-virulent S-INDEL and high-virulent non-S-INDEL PEDV strains induce differential innate immune response pathways *in vivo*. In six-day-old piglets, S-INDEL PEDV infection induced proinflammatory cytokines through the non-canonical NF-κB signaling pathway via RIG-I receptor activation. However, non-S-INDEL PEDV infection suppressed proinflammatory cytokine secretion and type I IFN production by down-regulating TLRs and canonical NF-κB signaling pathway mediators [65]. Defining PEDV immune evasion strategies is integral for identification of key virulence factors that inform vaccine design.

Viral infections also recruit innate immune cells like NK cells, dendritic cells (DCs), and macrophages to the sight of infection. NK cells are responsible for the cytotoxic killing of virus-infected cells and play an important role in viral clearance [66]. Greater NK cell frequency, lysis activity, and IFN-γ production in both serum and ileum of PEDV-infected piglets coincided with lower viral shedding and onset of diarrhea [41]. Additionally, serum levels of cytokines IFN-α and IL-12 were induced later and at significantly lower levels in older weaned piglets compared with neonatal suckling piglets, indicating a greater ability to control infection. DCs are potent antigen presenting cells and also play a role in PEDV pathogenesis. For example, in piglets, monocyte-derived DCs (mo-DCs) expand upon PEDV infection *in vivo* [67] and alter cytokine responses *in vitro* [67,68]. DC-targeting approaches for PEDV vaccines generated neutralizing antibody responses and provided partial protection against PEDV infection [69,70]. However, there is contrasting evidence regarding the ability of PEDV to productively infect DCs [67,71]. A recent study by Li et al. demonstrated that PEDV can replicate in the nasal cavity and suggested that PEDV enters submucosal DCs near infected nasal epithelial cells [24]. From there, the authors purport that PEDV accessed migrating T cells, which when in peripheral blood traffic to the intestinal epithelium leading to PEDV infection of the gut [24]. However, it is possible that these pigs were inoculated via both intranasal (IN) and oral routes. Indeed, the minimum infectious dose of the emerging highly virulent PEDV strain was very low. For example only 1 PFU was required to induce 100% diarrhea in 4-day-old cesarean-derived, colostrum-deprived piglets [72]. Additionally, a TCID_50_ of only 0.56 was needed to induce 100% diarrhea in 5-day-old conventional piglets [73]. The pigs could have been intestinally infected by uptake of only a tiny portion (≥0.0001 µL) of the inocula that contained a high dose of PEDV (7 log10 PFU in 1 mL). Therefore, an experimental design that mimics natural airborne transmission with a very low amount of virus inocula in a very low volume or by blocking the inocula entering the esophagus after IN spray, is needed. Lastly, relatively low levels of PEDV replication can be detected in tissue-resident porcine macrophages *in vivo* [74,75,76] and in porcine alveolar macrophages *in vitro* [55]. Macrophages likely play a protective role against infection while serving as a reservoir for replicating virus.

It is important to note that the majority of studies investigating the innate immune response to PEDV were conducted in cell lines *in vitro* or in neonatal and weaned piglets *in vivo*. There is a paucity of information regarding innate immune mechanisms in PEDV-infected pregnant swine. In a recent study, pregnant gilts in their first trimester had higher NK cell lysis activity compared with second and third trimester gilts [77]. This suggests that stage of gestation and pregnancy-associated hormones may play a role in innate immune responses in pregnant swine. Innate immunity is not compromised during pregnancy in swine and humans, despite an increase in regulatory and Th2 cytokines like TGF-β, IL-4, and IL-10 in the second trimester [77,78]. Indeed, innate immune parameters like type I and II IFNs and acute phase proinflammatory cytokines are integral to the success of early pregnancy in swine and humans [79,80,81]. Additionally, studies of pregnant women suggest that innate immune cells are actually more responsive to stimuli than in nonpregnant women. In response to influenza antigen, pregnant women have a significantly greater percentage of MIP-1a-producing and polyfunctional NK cells and monocytes and DCs exhibited exaggerated proinflammatory immune responses compared with non-pregnant women [82,83]. Phagocytic activity, *α*-defensin expression, numbers of neutrophils, monocytes and DCs, and STAT1 signaling are all enhanced during pregnancy in humans, notably in the second and third trimesters [84,85,86]. A decrease in proinflammatory cytokines during pregnancy without compromising innate immune functions is likely an evolutionary adaptation to protect the mother against Th1-related pathogenic infections without rejection of the fetus. Research is needed to determine the role of innate immune responses to PEDV infection during pregnancy in swine as it will likely have important implications for vaccine design, adjuvant formulation and generation of maternal antibodies for neonatal piglet protection via lactogenic immunity.

### 3.2. Adaptive Immunity

**Figure 1:**
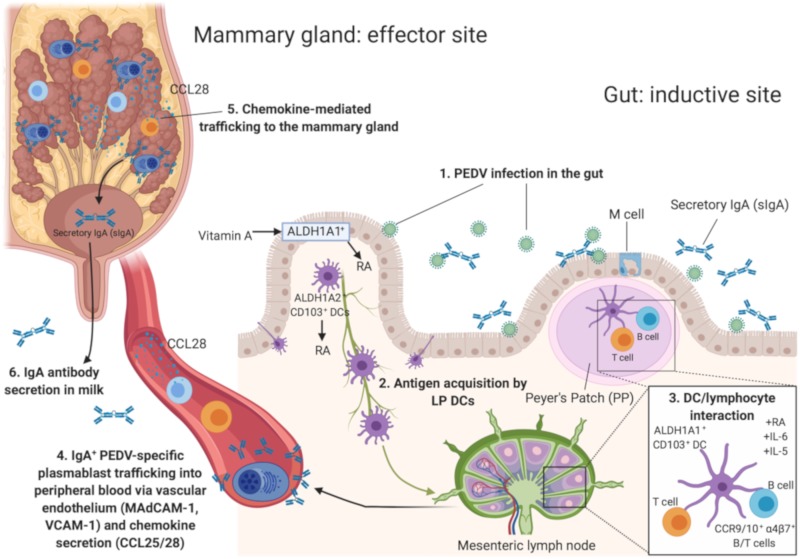
Schematic depicting the gut-mammary gland (MG)-secretory IgA axis (sIgA) and trafficking molecules in the gut and MG. After (1) porcine epidemic diarrhea virus (PEDV) infection in the gut, (2) lamina propria (LP) ALDH1A1/2^+^ CD103^+^ dendritic cells (DCs) acquire antigen from the gut lumen or within the subepithelial dome of Peyer’s Patches (PP) after transport by nearby M cells. (3) CD103^+^ DCs within both the PP and the mesenteric lymph node and in cooperation with retinoic acid (RA) and locally produced cytokines IL-6 and IL-5, upregulate mucosal trafficking adhesion molecules integrin α4β7 and chemokine receptor 9 (CCR9) expression in the intestine. (4) IgA^+^ plasmablasts migrate out of secondary lymphoid tissue and into the periphery by binding receptors (i.e. MAdCAM-1 and VCAM-1) on vascular endothelium. IgA^+^ plasmablasts reach the mammary gland by (5) chemokine ligand 28 (CCL28)-induced site directed migration. (6) Once in the mammary gland, IgA^+^ plasmablasts undergo terminal plasma cell differentiation and secrete IgA antibodies into milk.

Generating protective antibody responses in the gut is dependent on affinity matured and long lasting memory B cells in germinal centers of intestinal Peyer’s Patches [87]. To this end, DCs, T and B cells in gastrointestinal associated lymphoid tissue (GALT) must be exposed to viral antigens to promote antigen presentation and antibody production (Figure 1 [88]). 

DCs can sample luminal antigen directly by extending dendrites between intestinal epithelial cells or process viral antigen from apoptotic and dying intestinal epithelial cells during infection [89]. PEDV gut infection in piglets resulted in increased intestinal DC sampling and activation of T-cell proliferation *in vivo* [67]. Additionally, in a recent study, PEDV orally inoculated 5-day-old suckling piglets had significantly higher systemic CD8^+^ but not CD4^+^ T cell frequencies at 14 days post inoculation (DPI) compared with IN or intramuscularly (IM) inoculated piglets [90]. Considering CD8^+^ T cells are responsible for killing virus-infected cells [91], oral inoculation of PEDV may be ideal for generating PEDV-specific protective T cell responses in the gut that traffic to blood. Interestingly, Annamalai et al. demonstrated that 9-day-old PEDV-inoculated suckling piglets who had significantly higher diarrhea scores, earlier fecal PEDV RNA shedding, and significantly higher viremia, also had significantly greater CD4^+^ T cell frequencies in ileum compared to 26-day-old PEDV-inoculated weaned piglets [41]. However, ileal CD8^+^ T cell frequencies did not differ between the suckling and weaned piglets [41]. This suggests that a CD8^+^ T cell dominant CD4^+^/CD8^+^ ratio may be important for protection against PEDV. Few studies have investigated the role of CD4^+^ or CD8^+^ T cells during PEDV infection in pregnant swine. In one study, Subramaniam et al. sought to determine the immunogenicity of a DC-targeted PEDV S1 protein-based subunit vaccine. In vaccinated sows, the DC-targeted PEDV vaccine enhanced IFN-γ-producing CD4^+^ T cells and induced IgG antibodies in colostrum/milk. However, it did not mitigate PEDV clinical signs or decrease viral RNA loads in the intestines of PEDV-challenged piglets [92]. Interestingly, piglets born to sows given the DC-targeted PEDV vaccine had enhanced gross pathological lesions in the small intestine compared to PEDV-challenged piglets born to unvaccinated mothers [92]. The authors hypothesized that the induction of CD4^+^ T cells with a Th1 phenotype may have stimulated complement-activating IgG2 virus-specific antibodies that passively transferred to piglets and increased inflammation in the gut. This study highlights the need to better understand T cell immune responses during pregnancy and how they impact lactogenic immune protection in suckling piglets. 

In 7-week-old piglets experimentally orally inoculated with highly virulent PEDV, IgG antibody responses were detectable by 7–10 DPI with the majority of piglets seroconverting by 14 DPI. The IgG antibody response was specific for the S, N, and M structural proteins as well as whole virus particles [93]. The E protein did not generate a specific antibody response, likely due to the small amount that accumulates in infected cells combined with its low antigenicity [93]. While serum IgA levels were inconsistently measured in PEDV infection studies, de Arriba and colleagues demonstrated that serum PEDV IgA antibodies are detected in piglets experimentally infected with live virulent but not attenuated PEDV [94]. Additionally, there was a strong positive correlation between protection against PEDV and ASC levels detected in the gut lymphoid tissues and blood [94,95]. This is likely due to local viral stimulation of gut-derived ASCs and their trafficking into blood followed by secretion of PEDV antibodies [94,95]. PEDV IgA was also detectable in oral fluid and feces and may be correlated with piglet protection against PEDV [96,97,98]. Therefore, route of inoculation is important for generation of PEDV-specific IgG and IgA antibodies. PEDV-specific IgA antibody responses dominated in both systemic and intestinal compartments in oral PEDV inoculated piglets. Although IN inoculation induced circulating PEDV IgA antibody responses [90], little to none were detectable in the intestine, mimicking the responses of piglets inoculated IN with porcine respiratory coronavirus (PRCV) [99]. This demonstrates that PEDV and TGEV replication in the gut is required for induction of intestinal IgA antibody responses. As expected, PEDV IM inoculation resulted in strong serum IgG antibody responses but no PEDV-specific IgA antibodies were detected in serum or the intestine [90], again reflecting similar responses to TGEV [99]. 

Despite the ability of piglets to generate PEDV neutralizing IgA and IgG antibody responses [72], two main issues remain for direct vaccination of neonatal piglets against PEDV: (1) maternal antibodies in milk may interfere with live oral vaccines through neutralization and inhibition of B cell responses by epitope masking [100]; and (2) piglets need approximately three weeks to generate peak endogenous antibody production [101]. Consequently, induction of protective PEDV-specific IgG and IgA antibodies during pregnancy that are passively transferred to suckling piglets via the gut-MG-sIgA axis is required for immediate protection against PEDV. The following section will focus on the adaptive immune response in pregnant and lactating swine for generation of lactogenic immune protection against PEDV in suckling piglets.

### 3.3. Generating PEDV Lactogenic Immune Protection

Several key immunologic findings regarding generation of protective antibody response to enteric viruses in swine originated from our early work studying TGEV vaccines [14,102]. A high rate of protection to TGEV was achieved in suckling piglets when sows were orally infected with live virulent virus but not when they were inoculated parenterally [103,104,105]. Parenterally inoculated sows, however, had mainly TGEV-specific IgG antibodies in colostrum that declined rapidly in milk and provided little to no protection unless present in very high titers in colostrum [103,104,105]. Studies addressing the generation of protective PEDV-specific antibodies have been conducted in pregnant sows. For example, seropositive sows from a herd naturally infected with a ‘mild’ S INDEL strain of PEDV were re-exposed to a more virulent PEDV isolate (Genbank accession KF267450) toward the end of gestation. All piglets born to PEDV seropositive sows and challenged with the virulent PEDV isolate survived while piglets born to the naïve sow group had a 33% mortality rate [106]. Liu and colleagues also found that the piglets derived from two PEDV-field exposed-recovered sows did not have clinical signs after challenge with high doses of a virulent US PEDV strain PC22A [72]. These studies demonstrate that sows exposed to a US PEDV isolate could produce protective lactogenic immunity. More recent studies have investigated the impact of lactogenic immune protection of PEDV-challenged piglets in a sow infection model. Third trimester pregnant gilts given a higher dose of virulent US PEDV strain PC22A had increased virus neutralizing antibody titers in MG secretions and enhanced piglet protection compared with gilts given a lower dose [2]. Additionally, Poonsuk and colleagues demonstrated that piglets receiving milk with higher IgA antibody titers had significantly decreased fecal viral RNA shedding, providing additional evidence for the role of IgA antibodies in lactogenic immune protection against PEDV in neonatal piglets [107]. Work from the same group also suggested that systemic antibodies may be involved in PEDV protection. Indeed, piglets positive for circulating PEDV antibodies after intraperitoneal (IP) injection returned more quickly to normal body temperature and had decreased mortality post-PEDV challenge (USA/IN/2013/19338E) compared with piglets who were PEDV seronegative. Despite this, other measures of protection like PEDV fecal shedding, growth rates, and humoral immune responses were not improved by IP administration of PEDV-specific antibodies [108]. This suggests that while systemic antibodies may contribute to PEDV clearance, protection of neonatal piglets can be attributed mostly to maternal sIgA antibodies, as demonstrated in multiple studies [2,77,107].

Currently, there is limited data on PEDV antibody responses to the S INDEL variant in pregnant sows. In a recent paper, investigators used two European PEDV strains (PEDV-EU) to induce maternally-derived antibodies in sows and to assess lactogenic immune protection in piglets. Two sows received a cell culture-adapted PEDV strain (S INDEL) and another two sows were infected with field material from German PEDV outbreaks (S INDEL strain). All S INDEL-infected sows had PEDV IgG antibodies in serum and colostrum the day of farrowing. Milk PEDV IgA antibody levels were either negative or low and decreased throughout the study. All but two piglets born to S INDEL-infected sows were PEDV IgG antibody seropositive prior to S INDEL challenge with the corresponding PEDV inoculum at 3–6-days-of-age. Piglets born to S INDEL-infected sows had significantly decreased fecal viral shedding and diarrhea compared with those from naïve sows post piglet challenge [109,110]. While more studies are needed to compare maternal cross-protection between non-S INDEL and S INDEL strains, we can draw conclusions from infection models using suckling piglets and their contact exposed dams. Four litters of 3–4-day-old suckling piglets were inoculated using an S INDEL PEDV (Iowa 106) strain while two additional litters were inoculated with a non-S- INDEL PEDV (PC21A) strain and mock, respectively. At 21–29 DPI all piglets were challenged with the highly virulent original US PEDV PC21A strain. The S INDEL PEDV infection provided partial cross-protection to piglets against the non-S INDEL PEDV challenge. Piglet serum virus neutralizing and IgA antibody titers and ileal IgA ASC numbers were positively correlated with partial protection [32,111]. Additionally, infection of piglets resulted in contact exposure of the sows through piglet virus shedding. Both virus neutralizing and PEDV IgA antibody responses were detected in milk. However, milk virus neutralization antibody titers were lower in the S INDEL contact exposed sows compared with those of the non-S INDEL PEDV-exposed sows [32,111]. This suggests that differences observed in milk virus neutralizing antibody titers may contribute to protective efficacy in PEDV inoculated piglets.

### 3.4. Inducing Lactogenic Immunity against PEDV—Vaccination Strategies

Maternal vaccination strategies against enteric viruses that induce protective lactogenic immunity have been reviewed previously [2,3,14,102,112]. Different efforts towards initiating the gut-MG-sIgA axis during pregnancy with attenuated, subunit or recombinant vaccines have yielded varying results. To determine if a recombinant parapoxvirus [Orf virus (ORFV)] expressing the full-length PEDV S protein was immunogenic and generated protective lactogenic immunity in pregnant PEDV seronegative swine, primiparous gilts were assigned to one of three vaccine treatment groups: an unvaccinated negative control group (group 1), an ORFV-PEDV-S IM- immunized group (10^7.38^ TCID_50_) (group 2) or an ORFV-PEDV-S IM-immunized (10^7.38^ TCID_50_)/live PEDV-exposed (1 × 10^5^ TCID_50_) positive control group (group 3). Group 2 and 3 gilts were immunized IM 3X during pregnancy at gestation days 58, 79 and 100. In addition, group 3 gilts were exposed to live PEDV orally at gestation day 89. Piglets were PEDV-challenged at 7 days of age (2.5 × 10^2^ TCID_50_). PEDV IgG, IgA and virus neutralizing antibodies were detected in the serum of all piglets born to immunized gilts, demonstrating passive transfer of antibodies from milk. Group 3 gilts had the highest levels of serum PEDV IgG, IgA and virus neutralizing antibodies prior to parturition corresponding to the highest levels of PEDV IgG antibodies in colostrum and PEDV IgA antibodies in milk. Group 2 gilts had intermediate levels of protective antibodies in serum and milk associated with increased clinical scores in group 2 compared with group 3 PEDV-challenged piglets. Additionally, group 2 piglets had modestly decreased mortality in PEDV-challenged piglets (5% mortality) compared with group 3 piglets (0% mortality). In contrast Group 1 PEDV-challenged piglets received no PEDV antibodies in milk and had 50% mortality [113]. This demonstrates that IM PEDV subunit vaccines can elicit protective lactogenic PEDV antibodies of varying efficacy but do not provide complete protection against PEDV challenge in suckling piglets. This is likely because a PEDV subunit vaccine given IM does not initiate the gut-MG-sIgA axis during pregnancy such that PEDV sIgA antibodies do not accumulate in milk.

Due to the inability of PEDV IM vaccines to induce complete protection via lactogenic immunity, oral priming of seronegative sows with LAVs is a promising vaccination strategy against highly virulent PEDV [114]. However, increasing evidence demonstrates that vaccinating piglets with virulent PEDV provides enhanced protection against subsequent homologous challenge compared with vaccinating with attenuated strains [98,115,116]. This is likely because sufficient intestinal mucosal stimulation via PEDV replication is required for generation of protective neutralizing antibodies. Additionally, due to the potential of point mutations and recombination of PEDV strains in the field, there are major safety concerns of LAVs. Therefore, development of LAVs will require rationally introduced mutations in multiple locations that reduce the potential for reversion to virulence, while at the same time targeting genes that are not essential for viral replication and inducing protective immunity so that the mutant virus retains its immunogenicity. Indeed, efforts using reverse genetics technology for the rational design of PEDV LAV candidates have been recently reviewed [114]. For example, a recombinant PEDV vaccine candidate (KDKE^4A^-SYA), whose 2’-O methyltransferase activity of the nsp16 protein and endocytosis signal YxxΦ motif in the cytoplasmic tail of the S protein were inactivated, did not cause severe diarrhea and mortality in 4-day-old gnotobiotic piglets, and successfully protected 100% of piglets from diarrhea post challenge with a high dose (6 log_10_ PFU per pig) of highly virulent PEDV three weeks later. No reversions of the introduced mutations were observed after three passages of KDKE^4A^-SYA in pigs [56]. Additionally, a TGEV-PEDV chimeric virus vaccine (rTGEV-RS-SPEDV) induced partial protection in PEDV-challenged 21-day-old piglets [117]. These vaccine candidates were designed to contain attenuating mutations in several locations of the viral genome to control for vaccine virus reversion to virulence. These studies demonstrate that reverse genetics is a useful platform for development of LAV candidates with enhanced safety profiles when compared with traditionally attenuated vaccines that contain non-controlled mutations throughout the genome. However, considering the substantial differences in immune responses in neonatal piglets compared with pregnant swine, further experiments are required to determine the efficacy of these vaccine candidates during pregnancy. Using oral prime/parenteral booster vaccine approaches is also a promising strategy for generating protective lactogenic immunity. In a field study, PEDV seropositive sows given an IM injection of a conditionally licensed, adjuvanted inactivated PEDV vaccine had increased neutralizing antibody titers and anti-PEDV IgA and IgG antibodies in colostrum and milk [118]. However, because this study was on a commercial swine farm, piglets were not PEDV challenged. In pregnant sows orally primed with an S DEL5/ORF3 live vaccine and boosted twice IM with a commercial killed vaccine prior to parturition, complete lactogenic immune protection was induced after virulent G2b challenge of the neonatal piglets [119]. In another study, pregnant sows were orally administrated PEDV-loaded microspheres and boosted with a heterologous inactivated PEDV 21 days later [120]. Over 90% of the PEDV-challenged piglets born to vaccinated sows were protected. It is likely that prime/boost strategies will be required for the sustained generation of protective maternal antibodies, particularly if the animal was primed prior to conception [120].

There is increasing interest in identifying vaccine adjuvants that effectively induce mucosal immune responses. As previously described, DC-targeted approaches have been developed to enhance mucosal delivery efficiency of PEDV antigens [69,92]. Recently, Wang and colleagues generated a recombinant *Lacotobacillus* (*L393*) to secrete a DC-targeted peptide conjugated to PEDV antigen. After oral vaccination in neonatal piglets, this probiotic vaccine (*pPG-COE-DCpep/L393)* elicited PEDV-specific IgA and IgG antibodies and IFN-γ-producing CD4^+^ T cells [70]. These results are similar to those described earlier where sows vaccinated with a DC-targeted PEDV vaccine had increased IFN-γ-producing CD4^+^ T cells (Th1 phenotype). However, *pPG-COE-DCpep/L393* will need to be evaluated in a pregnant and lactating sow model as increased IFN-γ-producing CD4^+^ T cells after DC-targeting PEDV vaccination of sows resulted in increased gross pathology in the intestines of PEDV-challenged piglets compared with piglets born from unvaccinated sows [92]. This demonstrates the importance of using sow models when developing PEDV vaccines aimed to protect suckling piglets.

There are still unanswered questions regarding the mechanisms of the gut-MG-sIgA axis and how it relates to immunization timing during pregnancy and immune status. A recent study demonstrated that gestational age at the time of PEDV infection is important for the development of protective lactogenic immunity [77]. Gilts who were PEDV infected in their second trimester had significantly higher PEDV-specific IgA and IgG antibodies in serum and in milk compared with first and third trimester PEDV infected gilts. This resulted in 100% lactogenic immune protection in PEDV infected piglets. Additionally, piglet protection correlated with increased PEDV IgA ASCs, IgA antibodies, virus neutralizing antibodies in milk and PEDV IgA and IgG antibodies in piglet serum [77]. Likely the differences in PEDV immune responses between trimesters were attributable to the dynamic changes in circulating plasma hormone levels in swine. Understanding MG immunology, mechanisms of the gut-MG-sIgA axis and how pregnancy-associated hormones influence the immune response in pregnant and lactating swine is critical for the development of successful maternal immunization strategies against PEDV.

## 4. Host Factors Impacting Maternal Immunity in Swine

### 4.1. Basic Aspects of Lymphocyte Mucosal Trafficking

Pathogen-specific immunity in colostrum/milk is dependent on antigen-stimulated IgA^+^ plasmablasts (immature plasma cells) migrating from the intestine to the MG and sIgA antibodies accumulating in milk [104,121,122]. Therefore, understanding the host factors involved in the gut-MG-sIgA axis during enteric viral infection is imperative for the development of maternal immunization strategies. The basic aspects of lymphocyte mucosal trafficking in swine have been reviewed elsewhere [2,123]. Briefly, naïve lymphocytes migrate out of the bone marrow (B cells) and thymus (T cells) and continuously circulate between blood and secondary lymphoid tissues [124]. After encountering an antigen, stimulated B and T cells migrate to extralymphoid tissues, like the lamina propria and MG, by crossing specialized blood vessels known as high endothelial venules (HEVs) [125]. B and T cell extravasation through HEVs is a multistep process involving complementary homing ligands and receptors on lymphocytes and HEVs [126,127]. Lymphocyte adhesion molecule interactions with HEV ligands cause cells to tether and roll along HEV surfaces. In addition, chemoattractant receptors must be activated by chemokines to induce integrin-dependent docking of lymphocytes under shear flow followed by diapedesis of the cells through HEVs into tissue microenvironments [128,129].

Differential expression of homing molecules on lymphocytes and HEVs as well as expression of chemoattractant receptors and chemokines facilitate peripheral versus mucosal homing. Distinguishing mechanisms between mucosal and peripheral lymphocyte homing is important for designing vaccines and adjuvants meant for mucosal pathogens. For example, the interaction of cellular ligands L- selectin, α_4_β_1_, and chemokine receptor (CCR)7 with peripheral node addressin (PNAd), vascular cellular adhesion molecule 1 (VCAM-1), and chemokine ligand (CCL)21, respectively, controls lymphocyte migration to peripheral tissues. However trafficking in the intestine is largely regulated by lymphocyte expression of α_4_β_7_ interacting with mucosal addressin cellular adhesion molecule 1 (MAdCAM-1) on HEVs in GALT [130,131]. Chemokine ligands CCL25 and CCL28 on the surface of endothelial cells are involved in intestinal lymphocyte trafficking by binding lymphocyte chemokine receptors, CCR9 and CCR10, respectively. Interestingly, lymphocyte homing to the MG is dependent on the CCR10/CCL28 interaction [132,133,134,135]. In mice, humans and swine, CCR10 is selectively expressed by IgA ASCs. In mice given a CCL28-blocking antibody and in CCR10-deficient transgenic mice, IgA ASCs failed to migrate to the MG and milk of lactating animals, demonstrating the necessity of the CCR10/CCL28 interaction for the gut-MG-sIgA axis.

### 4.2. Role of Pregnancy-Associated Hormones and Gestational Stage in Lymphocyte Mucosal Trafficking

As described above, α_4_β_7_, CCR9, and CCR10 expressed on the surface of lymphocytes interact with MAdCAM-1, CCL25, and CCL28 on endothelial cells, respectively and regulates lymphocyte migration in and out of extralymphoid tissues. However, CCR10 expression on migrating IgA^+^ plasmablasts interacting with CCL28 on MG endothelial cells has been shown to be integral to the regulation of IgA^+^ ASCs to the MG. Therefore, identifying variables during pregnancy that influence homing marker expression and lymphocyte trafficking is imperative for developing gestational vaccines that enhance lactogenic immunity. Early studies demonstrated that pregnancy associated hormones may influence lymphocyte migration from the intestine to the MG [136]. Administration of estrogen (E2), progesterone (P4), and prolactin (PRL) in ovariectomized mice increased mesenteric lymph node (MLN)-derived IgA^+^ plasmablast trafficking to the MG. This study has not been replicated in swine, however, associative evidence exists between hormone levels and lymphocyte trafficking to the MG. In swine, mRNA of CCL28 is upregulated in the MG at late gestation and during lactation and the CCL28 protein is secreted into milk, supporting the hypothesis of CCL28’s chemoattractant role in the porcine MG [137,138,139].

**Figure 2:**
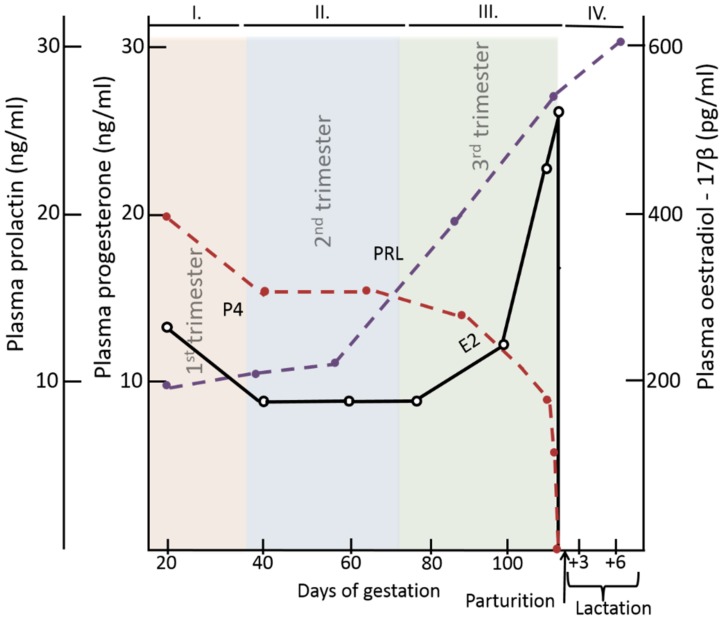
The relative concentrations of progesterone [(P4) red dashed line], estradiol [(E2) black line] and prolactin [(PRL) purple dashed line] in swine circulation.

**Figure 3:**
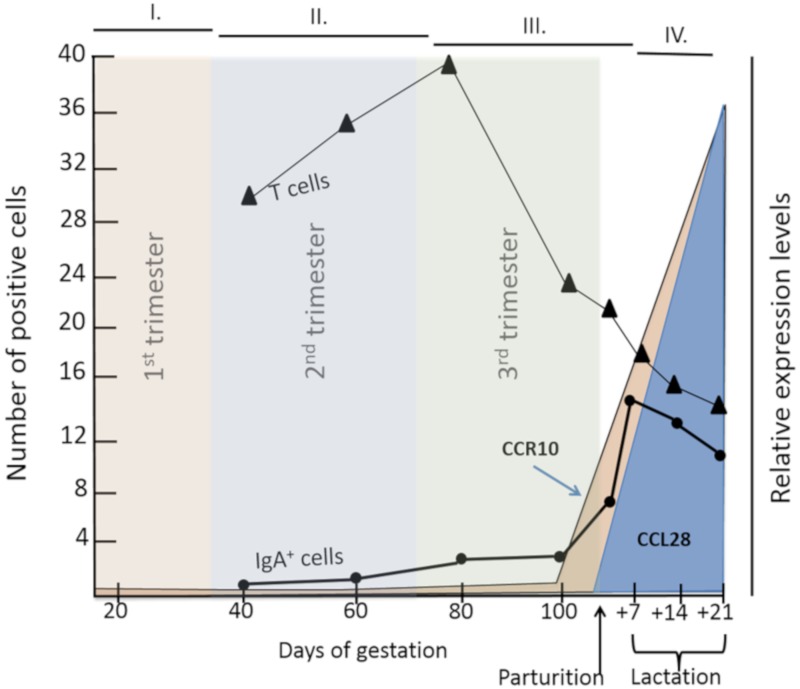
Schematic of relative T and IgA+ B cell numbers and chemokine CC receptor (CCR)10 and chemokine CC ligand (CCL)28 relative expression in the swine mammary gland (MG). Adapted from Chabaudie et al., 1993, Meurens et al., 2006, Bourges et al., 2008.

During pregnancy, a gilt or sow will experience dynamic changes in circulating hormone levels (Figure 2). In swine, P4 dominates in the first trimester (Figure 2, period I.), E2 dominates in the third trimester (Figure 2, periods III.), and PRL increases in the third trimester and peaks during lactation (Figure 2, periods III. and IV.), respectively [140,141,142]. The increase in E2 and PRL concentrations in the third trimester corresponds with an increase in expression of CCR10 and CCL28 and number of IgA^+^ cells in the MG (Figure 3 [143,144]). Alternatively, the change in T cells in the MG is not associated with decreasing P4 or increasing E2 or PRL plasma levels, suggesting that E2 and PRL impact IgA^+^ plasmablast homing (Figure 3). Currently, there is no research demonstrating a role for E2 or PRL directly in swine. However, based on the increase of E2 and PRL in the third trimester, combined with a concurrent increase in CCR10 and CCL28 expression and IgA^+^ cells, it is likely that there is a window of time during gestation in which swine lymphocytes are more responsive to pregnancy-associated/mammogenic hormones. Elucidating the optimal time(s) to vaccinate a gestating female should enhance oral vaccines protocols, improving animal health, and reducing costs for swine producers.

To further understand the role of pregnancy-associated hormones on the gut-MG-sIgA axis in swine, it is also important to review how these hormones influence immunity in other species. For example, starting in the second trimester to the early post-partum period, pregnant women are more susceptible to severe influenza when compared with non-pregnant women [145,146]. The heightened risk of infection during pregnancy drove earlier hypotheses that pregnancy is a state of immunological suppression. However, recent research suggests that pregnancy is a state of both pro- and anti-inflammatory immune responses, depending on the stage of pregnancy [147]. For example, in malaria-endemic regions, pregnant women are more susceptible to malaria infection during the first half of gestation and their risk of infection decreased during the second half [148]. The risk of death from Lassa fever, caused by the Lassa virus, is higher in the third trimester compared with the first two trimesters [149]. This evidence demonstrates that pregnancy should not be evaluated as a single event. Pregnancy could be more accurately defined as three immunological phases characterized by distinct hormone and immune responses (Figure 2 and Figure 3) [147]. A recent study of pregnant swine demonstrated that gestational stage at time of PEDV infection is integral to the development of protective lactogenic immunity [77]. It is likely that the extreme changes in hormones throughout pregnancy (Figure 2) contribute to the differences in pathogen susceptibility among trimesters and have implications for vaccine design.

### 4.3. Other Factors

Micronutrients like vitamin A (VA) have pleiotropic effects on the immune system (Figure 1) [150,151]. Evidence of intestinal dysbiosis and an increase in disease susceptibility in VA deficient humans and animals suggested that VA is a critical mediator of intestinal mucosal immune responses [151,152,153]. VA is required for imprinting gut homing phenotypes on B and T lymphocytes by GALT-associated DCs and retinoic acid (RA) synthesizing enzymes [retinaldehyde dehydrogenase (RALDH)-1, 2 and 3] (Figure 1) [154,155]. A recent study demonstrated that oral vitamin A supplementation (30,000 IU/day) of gilts during mid-pregnancy through lactation increased PEDV IgA ASCs and PEDV IgA antibodies in serum prepartum and IgA^+^β7^+^ (gut homing) cells in milk post piglet challenge in the PEDV-infected gilts. While not significantly different, the survival rate of PEDV+VA litters was 74.2% compared with 55.9% in PEDV litters [156]. These findings suggest that VA may act as an adjuvant during pregnancy to enhance lactogenic immunity to enteric viral infection (Figure 1). 

Similarly, there is associative evidence for vitamin D as an antiviral factor for many different viral infections including influenza, HIV, and cytomegalovirus [157]. Recent data demonstrated that weaned piglets supplemented with 25-hydroxyvitamin D_3_ (155.5 µg/day) had decreased intestinal damage and downregulated inflammatory cytokines and interferon signal pathway-related genes [158]. Currently, there are no studies investigating the role of vitamin D supplementation in pregnant swine. Future studies are needed to better understand the role of micronutrients, like vitamin A and vitamin D, on generation of immune responses to PEDV in pregnant swine or to other enteric viruses in pregnant swine and humans.

Parity may also play a role in lactogenic immune protection against PEDV in suckling piglets. In a recent study investigating the prevalence of rotavirus C (RVC) in a US commercial swine herd, multiparous sows farrowed healthier litters with a significantly decreased prevalence of diarrhea. This corresponded to higher RVC IgG and IgA antibody titers in milk but no differences were observed in serum [159]. In another study, multiparous sows had greater neutralizing activity in serum against swine influenza viruses than primiparous gilts but milk antibodies were not measured [160]. It is likely that for enteric viruses like RV and PEDV, locally produced IgA ASCs and antibodies secreted in the gut traffic to the MG via the gut-MG-sIgA axis and contribute to piglet protection against clinical disease [1,2,3]. Similar studies investigating the role of parity in generation of lactogenic immunity to PEDV are necessary because different strategies may be needed to boost piglet protection in multiparous vs. primiparous lactating swine.

## 5. Conclusions

Since the re-emergence of PEDV in the US in 2013, considerable efforts have focused on developing a PEDV vaccine that generates immune protection in susceptible piglets. However, safe and effective vaccines against highly virulent PEDV strains are still unavailable. It is likely that an effective LAV will need to target gestating and lactating swine to stimulate the passive transfer of protective IgA antibodies in milk via the gut-MG-sIgA axis. This is especially true due to low endogenous antibody production in piglets during the first two weeks of life. Despite this, most of the studies on the role of host immune factors on PEDV pathogenesis have been conducted in piglet infection models. Considering the substantial differences in immune responses between neonatal piglets and gestating/lactating sows, concerted efforts are required to shift towards developing LAVs using pregnant and lactating swine models to induce passive immunity in piglets. Indeed, many unanswered questions remain regarding host immune factors during pregnancy that may influence the induction of the gut-MG-sIgA axis including gestational age at time of inoculation, pregnancy-associated hormones, lymphocyte trafficking kinetics, parity, and micronutrients in the diet. For example, our PEDV infection studies using a pregnant model demonstrated that second trimester gilts have the greatest capacity to generate a robust PEDV humoral immune response prior to parturition, resulting in the highest amount of lactogenic immune protection against PEDV challenge in neonatal suckling piglets [77]. Our findings suggest that pregnancy should not be evaluated as a single event and development of a successful PEDV LAV requires consideration of stage of gestation at the time of vaccination. Additionally, we demonstrated that oral VA supplementation can be used as a cost-effective strategy to stimulate and enhance the gut-MG-sIgA axis to increase lactogenic immune protection of neonatal suckling piglets [156]. While studying PEDV immunity in pregnant and lactating sows is not trivial due to the associated increased labor and costs, it is critical for the development of LAVs that protect against PEDV in neonatal suckling piglets. Additionally, a pregnant swine model is applicable for the investigation of passive immune responses to other endemic and emerging enteric viral diseases in both humans and animals, as similar maternal vaccination strategies may be needed to enhance the gut-MG-sIgA axis and neonatal protection. Optimizing PEDV vaccine efficacy in pregnant swine to target the stage when intestinal lymphocytes are most responsive to mucosal trafficking will lead to enhanced lactogenic immunity and decreased morbidity and mortality in neonatal suckling piglets.
